# Characteristics and prevalence of musculoskeletal injury in professional and non-professional ballet dancers

**DOI:** 10.1590/bjpt-rbf.2014.0142

**Published:** 2016-01-19

**Authors:** Michelle S. S. Costa, Arthur S. Ferreira, Marco Orsini, Elirez B. Silva, Lilian R. Felicio

**Affiliations:** 1 Programa de Pós-graduação em Ciências da Reabilitação, Centro Universitário Augusto Motta (UNISUAM), Rio de Janeiro, RJ, Brazil; 2 Programa de Pós-graduação em Exercício e Ciência do Esporte, Universidade Gama Filho (UGF), Rio de Janeiro, RJ, Brazil; 3 Curso de Fisioterapia, Universidade Federal de Uberlândia (UFU), Uberlândia, MG, Brazil

**Keywords:** ballet dancers, prevalence, injuries, movement

## Abstract

**BACKGROUND:**

Ballet is a high-performance activity that requires an advanced level of technical skills. Ballet places great stress on tendons, muscles, bones, and joints and may act directly as a trigger of injury by overuse.

**OBJECTIVES:**

1) to describe the main types of injuries and affected areas related to classical ballet and 2) to compare the frequency of musculoskeletal injuries among professional and non-professional ballet dancers, considering possible gender differences among the professional dancers.

**METHOD:**

A total of 110 questionnaires were answered by professional and non-professional dancers. The questionnaire contained items related to the presence of injury, the regions involved, and the mechanism of the injury.

**RESULTS:**

We observed a high frequency of musculoskeletal injuries, with ankle sprains accounting for 69.8% of injuries in professional dancers and 42.1% in non-professional dancers. Pirouettes were the most frequent mechanism of injury in professional dancers, accounting for 67.9% of injuries, whereas in the non-professional dancers, repetitive movement was the most common mechanism (28.1%). Ankle sprains occurred in 90% of the women's injuries, and muscle sprains occurred in 54.5% of the men's injuries. The most frequent injury location was the ankle joint in both sexes among the professional dancers, with 67.6% in women and 40.9% in men.

**CONCLUSIONS:**

The identification of the mechanism of injury and time of practice may contribute to better therapeutic action aimed at the proper function of the dancers' bodies and improved performance by these athletes.

## Bullet points

- Pirouettes, repetitive movements, and landing jumps were the common mechanism of injury.
- Ankle sprains are the main injuries in professional and non-professional ballet dancers.
- Prevention programs should be conducted, with emphasis on exercises related to motor skills.

## Introduction

Dance is a series of movements in which the person moves in space and time to the rhythm of music. It is also defined as a specific expression of human motor behavior^1^. Ballet is a high-performance dance that requires an advanced level of technical skills. Ballet frequently places great stress on tendons, muscles, bones, and joints and thus may trigger injuries^2^. Ballet dancers are described as athletes because they can perform complex, physically demanding routines and are subjected to long periods of coaching^3^, currently being compared to top athletes^2^.

The need to understand the extent of injuries in dance has been a challenge due to methodological weaknesses involving the injuries and characteristics of the assessed individuals^3^. Sports literature has reports related to the prevalence and incidence of injuries, but there are few studies related to ballet^3^^-^ 5. Epidemiological studies of injuries in classical ballet indicate the length of performance as a major cause of injury, accounting for 40% to 80% of the lesions; however, these studies were based on amateur dancers and there was no control of the hours of daily practice^6^^-^ 8. In 1989, Bowling^4^ observed that professional ballet dancers had predominantly chronic injuries and that the cervical, lumbar, and ankle regions were the most affected, accounting for up to 2/3 of soft-tissue injuries. Most studies that investigated the incidence and prevalence of injuries in dance pointed to classical ballet as the dance modality with the highest technical demand^9^^-^ 11. Besides being the foundation for the performance of other forms of dance, it is the technique that has the highest rate of injuries^3^^,^ 12 ^-^ 19.

In classical ballet, the occurrence of ankle sprains by trauma has been reported as the most frequent injury. Picon et al.^20^ and Arendt and Kerschbaumer^21^ reported that these lesions are related to the movements in which the dancers remain in the tip position, when high loads on the joints are required, especially during jumping and landing. According to Picon et al.^20^ and Grego et al.^22^, approximately 86% to 97.48% of injuries in classical ballet are related to the lower limb, especially in the joints of the foot and ankle, and 64% of these lesions were caused by micro-trauma repetition^12^^,^ 20 ^,^ 22. Furthermore, according to Monteiro and Grego^7^, the knee and the hip correspond to 20% of regions with injury. Several factors are related to the onset and frequency of injuries in dance. However, muscle fatigue caused by overtraining, shows, and competitions seems to be one of the main triggering factors of injuries^12^^,^ 13 ^,^ 15.

Because of the high number of injuries suffered by dancers and the recognized need to direct attention to the involvements posed by the practice of ballet, the study of characterization and frequency of injuries becomes an important ally to health professionals, identifying the main mechanisms of injury, besides collaborating in methods of injury prevention in this population. Thus, this study aims to 1) describe the main types of injuries and affected regions related to classical ballet and 2) to compare the frequency of musculoskeletal injuries among professional and non-professional ballet dancers, considering possible gender differences among the professional ones.

## Method

### Sample

This was a retrospective descriptive study. One hundred and ten classical dancers were evaluated in action in the State of Rio de Janeiro, Brazil. The sample included 22 men and 88 women (17.6±9.3 years of professional experience in classical ballet), of which 53 were professionals and 57 were non-professionals. The study was approved by the ethics committee of the institution (CAAE: 0030.0.307.000-11, Centro Universitário Augusto Motta-UNISUAM, Rio de Janeiro, RJ, Brazil) and all ballet dancers signed a consent form after being informed of the design and objectives of the study.

### Questionnaire for prevalence of lesion

All participants were interviewed and completed a questionnaire containing items about how the injury occurred and its location. The items were selected from a self-administered questionnaire (Appendix A) based on Brooks et al.^23^^,^ 24 and Allen et al.^3^. Three hundred questionnaires in total were distributed in three schools and one ballet company in Rio de Janeiro, and in two schools and one ballet company in Niterói, RJ, Brazil. 110 questionnaires were returned completely answered.

### Statistical analysis

In the present study, the sample size was not calculated, and all of the 110 completely answered questionnaires were used. Descriptive data is presented using absolute and relative frequencies of categorical variables (gender; dominant body side; injury prevalence; types of injuries; location of injuries; mechanism of injury), grouped by involvement with ballet (professional and non-professional) and by gender in the professional group (male professionals and female professionals). Continuous variables (age; body mass index; hours/days of practice; years of practice; time off after injury) were presented as mean±SD because the null hypothesis of normality of distribution was not rejected using the Kolmogorov-Smirnov/Shapiro-Wilk test. For the comparative analysis, Student's t test and the Fisher test were conducted to compare averages and proportions, respectively, by adopting the value of p<0.05 as the significance level. The 95% confidence intervals were calculated to provide the precision of the statistical estimates. The software BioEstat version 5.3 was used for analysis.

## Results

### Professional versus non-professional ballet dancers

Anthropometric data and data from the training routine of male and female professional and non-professional dancers are described in Table 1. In the group of professional dancers, the mean age, hours of training, and practice time were higher than in the non-professional group. Regarding the professional group, the difference happens in the Body Mass Index (BMI) and years of practice, whereas the BMI was higher in male dancers and practicing time higher in females.

**Table 1.** Anthropometric data and data from routine of practices of professional and non-professional ballet dancers, as well as female and male professional dancers.

Group		Professional dancers		Non-professional	
				dancers	P-value
Subgroup	Men	Women	All	All	
Sample size	22	31	53	57	-
Age (years)^C^	34.1±7.1 [31.2; 37.1]	34.2±6.3 [32.0; 36.4]	34.2±6.6 [32.4; 35.9]	25.0±9.4 [22.5; 27.4]	<0.001^A^; 0.990^B^
Body mass index (kg/m^2^)^C^	23.6±1.1 [23.1; 24.1]	19.5±1.1 [19.1; 19.9]	21.2±2.3 [20.6; 21.8]	20.7±2.5 [20.0; 21.3]	0.250^A^; <0.001^B^
Right dominance^D^	19 (86.4) [72.0; 100.7]	28 (90.3) [79.9; 100.7]	47 (88.7) [80.1; 97.2]	55 (96.5) [91.7; 101.3]	0.151^A^; 0.683^B^
Practicing time (hours/day)^C^	5.8±0.9 [5.4; 6.2]	5.9±1.0 [5.5; 6.2]	5.8±1.0 [5.6; 6.1]	2.6±1.7 [2.2; 3.1]	<0.001^A^; 0.713^B^
Practice experience (years)^C^	18.1±5.4 [15.8; 20.4]	26.3±6.5 [24.0; 28.6]	22.9±7.3 [20.9; 24.9]	12.8±8.7 [10.6; 15.1]	<0.001^A^; <0.001^B^

Data shown as mean±SD or n (%) with 95% confidence interval [lower; upper]. AComparison between professional and non-professional ballet dancers. BComparison between women and men professional ballet dancers. CTwo-sample student's t-test, unequal variance (two-tailed). DFisher's exact test.

Regarding the most frequent types of injuries, ankle sprains correspond to 69.8% of injuries in the professional dancers and 42.1% in the non-professional ones, followed by muscle contractures in both groups. On the other hand, dislocations and subluxations were the least common lesions in both groups (Table 2).

**Table 2:** - Data about the kind and location of the lesion between professionals and non-professionals.

Group (n)	Professionals (n=53)	Non professionals (n=57)	P-value
Dancers with injuries caused by the dance	53 (100.0) [100.0; 100.0]	44 (77.2) [66.3; 88.1]	<0.001^B^
Time off after injury - days (mean / SD)	73.0±108.3 [43.8; 102.1]	26.1±71.1 [7.7; 44.6]	0.009^A^
Type of lesion Sprains	37 (69.8) [67.3; 82.2]	24 (42.1) [29.3; 54.9]	0.004^B^
Muscle contractures	19 (35.8) [33.2; 48.8]	17 (29.8) [17.9; 41.7]	0.546^B^
Others (e.g. muscle strain. direct trauma)	15 (28.3) [16.2; 40.4]	4 (7.0) [0.4; 13.6]	0.005^B^
Fractures	8 (15.1) [5.5; 24.7]	6 (10.5) [2.6; 18.5]	0.572^B^
Luxations	3 (5.7) [–0.6; 11.9]	3 (5.3) [–0.5; 11.1]	1.000^B^
Subluxations	3 (5.7) [–0.6; 11.9]	0 (0.0) [0.0; 0.0]	0.109^B^
Location of Lesions Ankle	30 (56.6) [43.3; 69.9]	20 (35.1) [22.7; 47.5]	0.035^B^
Knee	14 (26.4) [14.5; 38.3]	4 (7.0) [0.4; 13.6]	0.009^B^
Thigh and Leg	12 (22.6) [11.4; 33.9]	9 (15.8) [6.3; 25.3]	0.468^B^
Lumbar	12 (22.6) [11.4; 33.9]	3 (5.3) [–0.5; 11.1]	0.011^B^
Foot	8 (15.1) [5.5; 24.7]	7 (12.3) [3.8; 20.8]	0.783^B^
Shoulder	4 (7.5) [0.4; 14.7]	1 (1.8) [–1.7; 5.2]	0.194^B^
Hip and Pelvis	4 (7.5) [0.4; 14.7]	7 (12.3) [3.8; 20.8]	0.530^B^
Cervical	3 (5.7) [–0.6; 11.9]	1 (1.8) [–1.7; 5.2]	0.350^B^
Face	2 (3.8) [–1.4; 8.9]	0 (0.0) [0.0; 0.0]	0.230^B^
Elbow, forearm, wrist and hand	3 (5.7) [–0.6; 11.9]	0 (0.0) [0.0; 0.0]	0.109^B^
Thorax and abdomen	0 (0.0) [0.0; 0.0]	0 (0.0) [0.0; 0.0]	1.000^B^
Mechanism of injury Recurrence of injury	42 (79.2) [68.3; 90.2]	14 (24.6) [13.4; 35.7]	<0.001^B^
Spins (*pirouette*)	36 (67.9) [55.4; 80.5]	13 (22.8) [11.9; 33.7]	<0.001^B^
Repetitive movement	23 (43.4) [30.1; 56.7]	16 (28.1) [16.4; 39.7]	0.112^B^
Fall after jump	10 (18.9) [8.3; 29.4]	6 (10.5) [2.6; 18.5]	0.282^B^
Fall	3 (5.7) [–0.6; 11.9]	2 (3.5) [–1.3; 8.3]	0.671^B^

Data shown as mean±SD or n (%) with 95% confidence interval [lower; upper] (p

In relation to the location of the injury, the ankle corresponded to 56.6% in the professional dancers and 35.1% in the non-professionals. The knee was the second most affected region in the group of professional dancers, and the region of the hip and leg in non-professional dancers (Table 2).

Among the mechanisms of injury, the *pirouettes* were the most common in professional dancers, but in the non-professional ones, repetitive movements. However, the group of professional dancers had a higher frequency of this mechanism when compared to the non-professional group (Table 2).

In the present study, 36.4% (n=16) of the non-professional dancers and 83% (n=44) of professionals received some kind of physical therapy.

### Female versus male professional ballet dancers

All of the evaluated professional dancers, regardless of sex, had already suffered some injury as a result of ballet practice, however women have an average of post-injury off sport greater than men (p=0.009). Regarding the main types of injuries, sprains are more common in women (p=0.002) and muscle strains and sprains in men (p=0.001). The most affected region in both groups was the ankle, however women have a higher rate of injury than men in this region. Women have a high frequency of involvement in the knee, but no difference between groups was observed, since men have a high frequency of injury in the lumbar region when compared to women (Table 3).

**Table 3.** Data about the type and location of the lesion of professional female and male dancers.

Group (n)	Men (n=22)	Women (n=31)	P-value
Dancers with dancing injuries	22 (100.0) [100.0; 100.0]	31 (100.0) [100.0; 100.0]	1.000^B^
Time off after injury - days (mean / SD)	43.0±57.6 [19.0; 67.1]	94.2±129.9 [48.5; 139.9]	0.009^A^
Type of lesion Sprains	10 (45.5) [41.3; 66.3]	27 (87.1) [84.7; 98.9]	0.002^B^
Muscle contractures	11 (50.0) [45.7; 70.9]	8 (25.8) [22.7; 41.2]	0.088^B^
Others (e.g. muscle strain. direct trauma)	12 (54.5) [33.7; 75.4]	3 (9.7) [–0.7; 20.1]	0.001^B^
Fractures	2 (9.1) [–2.9; 21.1]	6 (19.4) [5.4; 33.3]	0.445^B^
Luxations	0 (0.0) [0.0; 0.0]	3 (9.7) [–0.7; 20.1]	0.258^B^
Subluxations	1 (4.5) [–4.2; 13.2]	2 (6.5) [–2.2; 15.1]	1.000^B^
Location of Lesions Ankle	9 (40.9) [20.4; 61.5]	21 (67.7) [51.3; 84.2]	0.091^B^
Knee	4 (18.2) [2.1; 34.3]	10 (32.3) [15.8; 48.7]	0.348^B^
Thigh and Leg	7 (31.8) [12.4; 51.3]	5 (16.1) [3.2; 29.1]	0.202^B^
Lumbar	10 (45.5) [24.6; 66.3]	2 (6.5) [–2.2; 15.1]	0.002^B^
Foot	1 (4.5) [–4.2; 13.2]	7 (22.6) [7.9; 37.3]	0.120^B^
Shoulder	2 (9.1) [–2.9; 21.1]	2 (6.5) [–2.2; 15.1]	1.000^B^
Hip and Pelvis	1 (4.5) [–4.2; 13.2]	3 (9.7) [–0.7; 20.1]	0.633^B^
Cervical	2 (9.1) [–2.9; 21.1]	1 (3.2) [–3.0; 9.4]	0.563^B^
Face	0 (0.0) [0.0; 0.0]	2 (6.5) [–2.2; 15.1]	0.505^B^
Elbow, forearm, wrist, and hand	1 (4.5) [–4.2; 13.2]	2 (6.5) [–2.2; 14.9]	1.000^B^
Thorax and abdomen	0 (0.0) [0.0; 0.0]	0 (0.0) [0.0; 0.0]	1.000^B^
Mechanism of injury Recurrence of injury	15 (68.2) [48.7; 87.6]	27 (87.1) [75.3; 98.9]	0.168^B^
Spins (*pirouette*)	9 (40.9) [20.4; 61.5]	27 (87.1) [75.3; 98.9]	0.001^B^
Repetitive movement	16 (72.7) [54.1; 91.3]	7 (22.6) [7.9; 37.3]	0.001^B^
Fall after jump	4 (18.2) [2.1; 34.3]	6 (46.2) [5.4; 33.3]	1.000^B^
Fall	2 (9.1) [–2.9; 21.1]	1 (3.2) [–3.0; 9.4]	0.563^B^

Data shown as mean±SD or n (%) with 95% confidence interval [lower; upper] (p

Among the mechanisms of injury, *pirouettes* were more frequent in women, while in men, repetitive movements were the most common mechanism (Table 3).

Among the professional dancers who had injuries due to the practice of ballet, 90% (n=27) of women and 91.3% (n=21) of professional men received some kind of physical therapy.

## Discussion

This study described the characteristics and frequency of injuries occurring in professional and non-professional ballet dancers, taking into consideration the characteristics and mechanisms of injury by sex and length of practice, in addition to the injured area.

The time in the profession with a company can provide challenges for ballet dancers, including the need to develop technical knowledge as well as achieve strength and required levels of technical execution. These demands may contribute to the increased rate of injuries in these ballet dancers compared to non-professionals ones^3^^,^ 25 ^,^ 26. Our data agree with these authors, because it was observed that professional ballet dancers with 5.6 hours of training/day presented higher frequency of injury in relation to the non-professional group with less training time.

Our data agree with Ekegren et al.^27^, who compared 266 elite ballet dancers with varied hours of daily training. These authors reported that, with increasing training duration, there was also an increased risk of injury, particularly due to overuse^27^. However, they did not assess the relationship between the training duration and region or type of injuries. Our data show the same behavior in professional ballet dancers in spite of the overall low frequency of injuries and high frequency of both sprains and muscle injury to the lower limbs. Altogether, these characteristics suggest that these regions are mainly subjected to overload, regardless of the training duration of dancers.

Like most sports, ballet is a strenuous activity and requires good athletic condition. However, in ballet, the movement is the final objective and requires exhaustive repetition, which increases the risk of musculoskeletal injury^17^^,^ 28. Due to the determination of ballet dancers, choreographers, and ballet masters to achieve "perfection" in the movements during training, many dancers suffer injuries prior to presentations and performances.

In the present study, we found a higher frequency of injury due to repetitive movements in professional dancers compared to non-professionals. In the group of professionals, men had a higher rate of injury with this mechanism because of the lifts and wide movements and jumps that they perform^29^. According to Guimarães and Simas^13^, both in professional and non-professional ballet dancers, the repetition of a particular part of choreography or isolated movement performed even after fatigue is responsible for several injuries.

Among the movements performed during training and performances, we highlight the *pointe*, *demi-pointe*, and *en dehors*. These positions can cause joint overload, as well as ligament and muscle micro-traumas, especially in the ankle and knee regions, increasing the likelihood of injury^30^, as observed in this study. Thus, our data strongly suggest the inclusion of exercise programs to prevent injury, including exercises to improve the muscle strength of the lower limbs as well as the back muscles, which are the most frequently affected regions.

The various *pointe* (tiptoe) positions in ballet vary according to the position of the feet and reduce the base of support, which requires great muscle and neurophysiological effort^30^. Added to these positions, the impact of falls after jumps and *pirouettes* is related to severe ligament and musculoskeletal injuries, especially to the lower limbs^31^.

The present study indicates that, among the regions affected in professional dancers, the ankle is the most common. In addition, professional women are the most affected. Our results agree with those observed by other authors; however, these authors reported about 86% of lower limb injuries, not discriminating the location of injuries in the lower limb^7^^,^ 9 ^,^ 13 ^,^ 20. Studies carried out in international dance companies confirm the results of this study, with ankle sprain as the highest prevalence of injuries in dancers^17^^-^ 19 ^,^ 32.

The frequency found for ankle sprains occurred in dancers aged 34.7±6.9 years of age. Paradoxically, in a study conducted in a Swiss ballet company, 75% of ankle sprains occurred in young ballet dancers under 26 years of age, showing that the more experienced the dancer is, the better the technique and the lower the risk of injury^17^. This fact was not observed in our study, in which 69.8% of sprains occurred in professional dancers with an average of 22.9 years of practice. These differences may be related to the presence of specialized medical teams in sport and the presence of injury prevention programs in ballet companies overseas, which does not happen in national companies. In addition, the specific gesture movements related to the practice of ballet should also be assessed to verify possible relationships between posture misalignments during performance and specific injuries.

Hillier et al.^19^ reported that men are more affected by trauma and women by repetitive stress, especially in the foot and ankle; however, our data do not agree with these authors, as professional women suffered more injuries during the *pirouette* when compared to men. Generally, the behavior of the characteristics and the frequency of the lesion between men and women are similar.

Our data indicate that, among professionals, 18.9% of the injury mechanisms are related to landing from jumps, with no differences between the sexes. As in the non-professional ballet dancers, injuries related to landing correspond to 10.5%. The jumps are highly complex movements that are performed most often by professional dancers.

Dore and Guerra^12^ evaluated musculoskeletal symptoms and observed a dominant prevalence of low back pain, followed by knee pain. Our results disagree with those reported by these authors, since we observed a low frequency of lumbar complaint among professionals. On the other hand, our data show the knee as the second affected region among the professionals. Guimarães and Simas^13^ described several factors that could contribute to the overloading of the knee joint, particularly improper training, repetitive jumps, spins, and the *plié* position, causing valgus knee and excessive hyperextension. The authors also reported that both the ankle joint and the knee suffer high stress during the practice of ballet.

Gamboa et al.^5^ also found a high frequency of ankle and knee injuries among professional ballet dancers, which is in agreement with our results. Moreover, these authors found an association between pronation of the subtalar joint and the prevalence of injury, overload to the knee, and risk of sprain of the anterior cruciate ligament. In spite of the homogeneity of our sample of professional ballet dancers, sports literature on this subject points to a large heterogeneity in type and location of injuries. Thus, ballet dancers should be screened with specific functional tests related to their performance, contributing to personalized prevention of musculoskeletal injuries.

Furthermore, our study showed that 90% of the female and 91.3% of the male professional dancers had undergone some kind of physical therapy, despite a high frequency of recurrence of injuries. Although we did not assess the kind of physical therapy treatment each dancer received, our data suggest the implementation of not only rehabilitative treatment, but also preventive treatment. Therefore, the therapist who is familiar with the prevalence and the mechanism of injury is better equipped to assess the type of injury involved and its extension and to design interventions that are more suitable for this type of athlete^3^.

The interpretation of the data from this study should take into consideration the absence of the sample size calculation; however, the specificity and technical quality of the sample should be considered. Moreover, the frequency of the injuries was determined according to the self-report of the dancers, which could affect comparisons between studies that considered the diagnosis given by expert professionals.

Based on these findings, we can conclude that non-professional and professional ballet dancers have a high frequency of musculoskeletal injuries, with the ankle being the most affected joint due to spinning and repetitive movements. We can also conclude that gender did not affect the frequency of lesions. Therefore, technical training sessions should be emphasized to promote more appropriate movements from the biomechanical point of view, which is expected to decrease the risk of injury by repetitive movements. Moreover, physical therapy follow-up should be considered in the rehabilitation process of ballet dancers to account for their specific motor skills (e.g. jumping, *pirouettes*) and to improve muscle function in body regions with increased risk of injuries (e.g. lumbar spine and lower limbs).

In summary, our data supports better therapeutic action in the intervention of injuries, such as prevention programs, taking into consideration the dancer's movements.
